# What Have We Learned in 30 Years of Investigations on *Bari* Transposons?

**DOI:** 10.3390/cells11030583

**Published:** 2022-02-08

**Authors:** Antonio Palazzo, Ruggiero Caizzi, Roberta Moschetti, René Massimiliano Marsano

**Affiliations:** Dipartimento di Biologia, Università di Bari, 70125 Bari, Italy; antonio.palazzo@uniba.it (A.P.); ruggiero.caizzi@gmail.com (R.C.); roberta.moschetti@uniba.it (R.M.)

**Keywords:** *Bari* transposons, Drosophila, regulation, transposon tandem repeat, horizontal transfer, blurry promoter, heterochromatin

## Abstract

Transposable elements (TEs) have been historically depicted as detrimental genetic entities that selfishly aim at perpetuating themselves, invading genomes, and destroying genes. Scientists often co-opt “special” TEs to develop new and powerful genetic tools, that will hopefully aid in changing the future of the human being. However, many TEs are gentle, rarely unleash themselves to harm the genome, and bashfully contribute to generating diversity and novelty in the genomes they have colonized, yet they offer the opportunity to develop new molecular tools. In this review we summarize 30 years of research focused on the *Bari* transposons. *Bari* is a “normal” transposon family that has colonized the genomes of several Drosophila species and introduced genomic novelties in the melanogaster species. We discuss how these results have contributed to advance the field of TE research and what future studies can still add to the current knowledge.

## 1. Introduction

Transposable elements are fundamental genetic units in the genomes of virtually all living organisms. TEs could be depicted as the characters of a happy ending fairytale. Initially regarded as “junk” and “useless”, TEs turned out to be considered as evolutionarily flagships after reconsidering the role they have had and still have in shaping genomes and their functioning. Moreover, the characterization of many transposition systems has led to the development of efficient DNA integration tools [1] as well as powerful genome engineering systems [2,3], and to the implementation of TE control regions into efficient expression systems [4].

It is a matter of fact that the hallmark of all TEs, i.e., the ability to integrate into chromosomes, is the most interesting aspect of TEs for many biologists, due to their many possible applications in a large group of fields in Life Sciences.

Efficient genome integration tools are indeed desirable to disrupt genes, either in a random or targeted way, and to introduce exogenous DNA into the preferred cellular or animal model. A special application of the latter practice is gene therapy, which consists in the introduction of the non-pathological allele in the affected cell type of a patient that suffers from a genetic-based disease, with the aim to rescue the illness phenotype.

In the past 40 years many transposition systems have been studied in detail with the aim to set up new and efficient DNA integration tools.

Historically, the Drosophila *P-element* was the first transposon-based transposition tool to be employed in functional genomics [5,6]. Unfortunately, its main limitation is the narrow host range of transposition [7], which makes it useless for much noble applications, such as gene therapy.

Elements of the *Tc1/mariner* are more tractable for this kind of application. *Tc1/mariner* elements belong to the Class II of the eukaryotic transposons and are widely distributed, from bacteria [8] to higher eukaryotes [9], with few exceptions. Their wide distribution in living organisms allowed the foundation of the *IS630/Tc1/mariner* superfamily.

More in general, the so-called “cut and paste” DNA transposons are the best candidates to develop molecular tools for transgenesis because of their simple mechanism of transposition and their poor requirement of host factor [10]. Two of these elements stepped into the limelight in the past decade, the *Sleeping Beauty* (SB) and the *piggyBac* (PB) elements [1]. SB is undoubtedly the most sophisticated transposon-based system in the context of therapeutic setup. The CARAMBA clinical trial (https://www.caramba-cart.eu; accessed 15 December 2021) currently uses an advanced SB-based transposon technology for therapeutic gene delivery [11].

However, not all the known transposition systems support this kind of application. Most known TEs have limited transposition performances (i.e., low transposition rate, narrow host specificity) or they have low flexibility (i.e., they are too large and complex or display low cargo capability) to allow the development of efficient genome integration systems. Nevertheless, many TEs are studied for their role in shaping genome structure [12] and gene expression [13] or to develop new and alternative technologies [3,4,14,15].

Among the *Tc1/mariner* superfamily of TEs the *Bari* family was discovered 30 years ago in the former Institute of Genetics at the University of Bari (Italy). Such discovery led to the foundation of a new research line, which is still currently under investigation, in a laboratory up to that time devoted to the study of glutamine synthetase [16,17,18].

In this review, we comprehensively summarize the results of 30 years of research that concern the Drosophila *Bari* elements, and frame these results in a comprehensive view in the field of TE research. We also provide more extensive hypotheses on the role of *Bari* transposons in the genome of Drosophilidae species.

### The Discovery of the Bari1 Transposon: An Historical Overview

The discovery of the *Bari* transposons occurred during the characterization of the h39 region of the mitotic chromosomes. This is a complex repetitive locus in *Drosophila melanogaster*, adjacent to the second chromosome’s centromere [19]. It was known from previous studies that mutant flies carrying the deletion of the h39 region showed a semi-lethal phenotype and low fitness [20]. The phenotype was associated with the deletion of the Responder satellite [21], the main satellite mapped in the h39 until then.

The team headed by Prof. Caizzi and Prof. Pimpinelli hypothesized that additional genetic and molecular entities could map in the h39 region, which could also account for the phenotype associated with the region deletion. In the main effort of characterizing the h39 region at the molecular level, they identified a novel repetitive sequence, uniquely mapping to this region. Originally, differential hybridization technique was used to identify, isolate, and subsequently clone h39-specific sequences. The existence of extraordinary genetic toolkits, such as precisely mapped chromosome rearrangements (the most effective was the Rsp^ins16^ (R16) deletion [22,23]) undoubtedly aided the genetic mapping in a heterochromatic region. Furthermore, the availability of molecular tools, such as the possibility to construct strain-specific genomic libraries, but most of all acrylamide gel electrophoresis to read Sanger sequencing reactions, strongly contributed to the characterization of a previously unknown sequence isolated from single copies dispersed in the euchromatin that was soon classified as a new transposon of the *Tc1/mariner* superfamily. It was named *Bari1* after its discovery in the Italian city of Bari, where the laboratory was based, and making the wish (which would later come true!) that other *Bari* elements might be discovered to complete the series.

**Figure 1:**
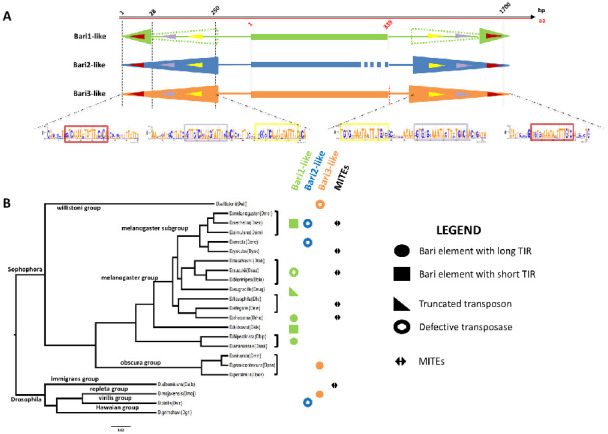
(**A**) Structure and TIRs organization of the three Bari sub-families. The length of the TIRs is indicated by colored arrowheads. *Bari1* elements may have two different TIR lengths (represented by solid and dashed green arrowheads, respectively). The three DRs (outer (red), middle (violet), and inner (yellow)) are shown as DNA logos [38] obtained by comparing at least 15 different Drosophila species. The elements represented in the picture (TIRs, ORFs) are not drawn to scale. (**B**) Distribution of *Bari*-like elements in the Drosophilidae (adapted from [30]). Symbols are explained in the figure legend and have the same color-code as in panel A (i.e., *Bari1*-like green; *Bari2*-like blue; *Bari3*-like orange).

*Bari1* is a DNA transposon belonging to the *IS639*/*Tc1*/*mariner* superfamily with 26 bp long inverted repeats (IRs) and three direct repeated sequences (DRs) [24], serving as the transposase binding sites (Figure 1A). As noted earlier in comparative studies, the 3xDRs structure is almost peculiar [25], since there are few known TEs with similar organization of the terminal sequences, including *Paris* [26], *S* [27], *minos* [28], and *SB* [29].

**Figure 2:**
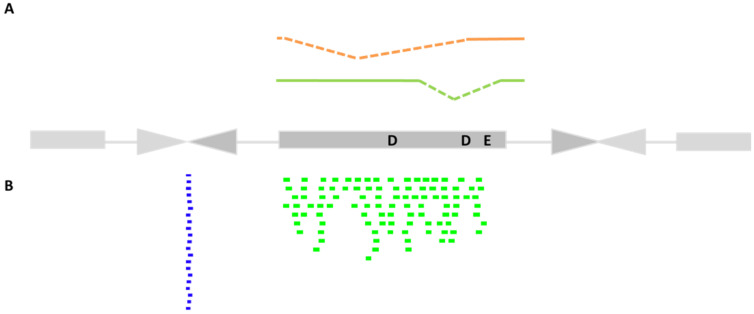
The regulation of *Bari* transposons. (**A**) Spliced transposase mRNA of *Bari1* (green) identified in [61] and *Bari3* (red) identified in [42]. Both spliced transcripts encode a transposase with non-functional catalytic domain. (**B**) Distribution of piRNA mapping to the *Bari1* transposon. The amount of piRNA is purely representative of the relative fraction matching the ORF (green) or the heterochromatic inter-monomer junctions (blue) of *Bari1* (see text for further details). A reference element.

If the identification of a new transposon in the early 1990s was *per se* a great advancement in the field of genome structure and evolution, the characterization of the peculiar arrangement of *Bari1* in a heterochromatic locus was breathtaking. The precise head-to-tail organization of roughly 80 *Bari1* copies in the h39 region of the mitotic chromosomes of *D. melanogaster* was featured by the systematic deletion of the very first two nucleotides in each copy. To our knowledge, this enigmatic organization still has no comparable described examples in the field of TEs. While other Drosophila species contain *Bari*-like transposons (see the Section “The Bari Family Grows Up”), the heterochromatic *Bari1* cluster seems to be specific to the melanogaster species. This organization suggests a recent-and species-specific evolutionary origin since it is specific to a single species. It also allows the discrimination of two sibling species (i.e., the melanogaster and simulans species) at the molecular level. Moreover, a second minor *Bari1* cluster has been recently described, which maps on the X chromosome of *D. melanogaster* [30,31]. This is a small cluster that is composed of six copies of *Bari1.* Surprisingly, both the main and the small clusters share the same di-nucleotide deletion at the 5′ end of each element, and the head-to-tail organization. In addition, both clusters share a heterochromatic localization, although on different chromosomes. In a retrospective view, it is worth noting that the presence of an additional *Bari1* tandem repeat was also evident since the 1993 paper. Indeed, in one of the Southern blot hybridization analyses presented in the paper, it was evident that the Rsp^ins16^ background still contains an additional *Bari1* sequence block arranged in a tandem repeat configuration (see Figure 4 in Caizzi et al., 1993 [32]. A similar pattern can be observed in Caggese et al., 1995 [33] (see Figure 2 therein), an observation that allows excluding technical artifacts. Taken together, these findings suggest a marked instability of the *Bari1* element in the melanogaster species, associated with an error-prone transposition that has occurred at least twice during the evolution [34]. An aberrant transposase activity that generated long concatemers form a circular transposon, in a way similar to the rolling circle replication mechanism, has been proposed to explain the origin of both the *Bari1* arrays [34]. However, the structural and functional role of these clusters (if any) remains currently unresolved.

Among the possible role of the *Bari* clusters, two scenarios can be envisioned. The simplest hypothesis is that it could have a regulatory function. The second hypothesis, which we are currently testing using transgenic strains, is that the *Bari* cluster could be involved in the structural organization of h39 domains, possibly aided by other repetitive sequences mapping in the same region [35,36,37].

## 2. The Bari Family Grows Up

### 2.1. Introducing Bari2 and Bari3

Studies aimed at the determination of the diffusion of the *Bari* transposons in Drosophila genus and in other Diptera species demonstrated that *Bari* transposons are widely represented in the Drosophila genus and that they can be subdivided into three distinct sub-families.

Former studies, mainly performed using Southern blot hybridization techniques, demonstrated the presence of homologous *Bari1* sequences in species closely related to *D. melanogaster* [39]. Weak or very faint hybridization signals suggested the presence of *Bari* elements that were divergent in sequence in distant species, belonging to the Sophophora and Drosophila sub-genera, and to the Zaprionus genus. Indeed, cloning and sequencing hybridizing fragments (showing weak hybridization signals) from *D. erecta* and *D. diplacantha* demonstrated the presence of another type of *Bari* element, which was named *Bari2*, in the genome of many species in which the *Bari1* element was also found. *Bari2* differs from *Bari1* in structure, since it is characterized by long TIRs (nearly identical 253 bp sequences) and no coding potential, due to numerous invalidating indels and frameshift mutations that disrupt its ability to encode a transposase (Figure 1A). Not a single active *Bari2* element has been found to date, making *Bari2* a non-autonomous sub-family. Notably, the distribution of *Bari2* in the genome of *D. erecta* is almost heterochromatic, suggesting either an insertion preference of the ancestral (active) form of *Bari2* or a recent elimination of the euchromatic copies.

The evolutionary link between the potentially active *Bari1* and the evolutionary knocked out *Bari2* elements was also difficult to understand. While the comparison of the ORF (and its protein product) of *Bari1* and the reconstructed consensus sequence of *Bari2* clearly suggested their evolutionary relationship, their TIRs are very dissimilar both in sequence and structure. *Bari2* is indeed featured by long TIRs, identical to each other. Only at the DRs level the two families share evident homology, a sign that both derived from an ancestral *Bari* transposon [24]. Why the TIRs of *Bari2* have been preserved in extant Drosophila species is not clear. An intriguing hypothesis, that parallelizes the one proposed for the human *Hsmar1* element, is that *Bari2* TIRs have been co-opted to titrate some endogenous nuclear protein [40,41] involved in the maintenance of the chromatin status or in chromatin remodeling or to titrate the transposase of active *Bari* elements.

The missing evolutionary link between *Bari1*, possessing short TIRs and intact ORF, and *Bari2*, possessing long TIRs and disrupted ORF, was subsequently identified with the discovery of the third *Bari* family in *D. mojavensis* [24]. *Bari3* was next identified in species of the obscura and the willistoni groups [30]. Many intact *Bari3* insertions and the polymorphism observed in geographically distinct populations suggested that it is an active transposon [42]. *Bari3* transposases share 80% similarity with *Bari1,* and it is featured by long TIRs with an IR/DR structure (Figure 1A) [24,42]. 

### 2.2. New Cognate Elements in New Species: The Crew Grows up in the Post-Genomic Era

The advent of the post-genomic era has offered the opportunity to perform comparative studies that were very difficult to perform without the availability of genome sequence assembly. Evidence of the presence of *Bari* elements in distant Drosophila species were mounting in a former work which identified homologous sequences in the Sophophora and Drosophila genera [39]. In a genomic survey study conducted in 23 species of Drosophila, several other elements related to the three known *Bari* sub-families were identified in newly sequenced Drosophila genomes (Figure 1B) [30]. In this study, *Bari*-like elements were identified and annotated in all but the *D. grimshawi* species. The extended annotation of *Bari*-like transposons suggests that, despite the diversity observed in the TIR structure, the DRs are well-conserved across *Bari* sub-families (Figure 1A) [24,30]. It is worth noting that an interesting *Bari1*-type element with long TIRs was identified in *D. rhopaloa* (Figure 1B), which further entangles the evolutionary dynamics involving the terminal ends of the *Bari* transposons.

The evolutionary scenario observed in the extant Drosophila species is complicated by the presence of an additional group of non-autonomous sequences called MITEs (Figure 1B). MITEs (Miniature Inverted repeats Transposable Elements) are frequently found in eukaryotic genomes [43,44,45,46] and they are considered as evolutionary byproducts, originating from a rearranged (i.e., internally deleted) ancestral form of TIR elements. Their subsequent amplification in the genome occurred through trans-complementation with the functional transposase expressed by active TEs. *Bari*-derived MITEs have been identified in *D. sechellia* [47] and in other Drosophila species [30]. *Bari*-derived MITEs can be categorized either as short- or long-MITEs, depending on their sequence length. While both forms share the same terminal sequences, the internal sequence can be highly variable in length, with the long form exhibiting sequences unrelated to *Bari* transposons [30]. It has been proposed that the TIRs of both functional and defective copies (including MITEs) that retain the transposase binding activity can act as buffer to titrate endogenous levels of the transposase, [40]. Therefore, it is possible that also *Bari*-derived MITEs are maintained for this regulatory purpose.

## 3. Horizontal Transfer Events Involving Bari Elements

Horizontal transfer (HT) is one of the most obscure yet fascinating aspects underlying the evolutionary dynamics of the genomes [48,49]. TEs are among the most prone DNA sequenced to take part in HT events [50,51]. While during the pre-genomic era horizontal transposon transfer (HTT) events could be only detected using molecular assays, currently we have potent bioinformatic tools that allow HTT inference supported by statistical methods [52]. It is now more evident from genome sequence comparison that HTT commonly occurs during evolution but it is hard to detect only for two basic limitations in our approaches. The first is intrinsic to the HTT process, in that we could detect it if occurs in germline cells. In this case, the horizontally transferred DNA can be transmitted in the population and we can detect it as an “alien” piece of DNA. Somatic HTT events could conversely result in a small number of mosaic organisms and would not be detected because of the low (or very low) representation of the transferred sequence in whole genome extracts. The second limitation in detecting HTT is due to the restricted number of sequenced genomes, compared to the number of extant species. Furthermore, even if there are more than 20,000 genome projects in NCBI (different advancement status—last access early December 2021) they refer to small individual samples, representative of entire populations or species. This gives us a poor vision of the sequence variability caused by HTT, with a consistent loss of event detection at the population level.

Several studies suggest that *Bari1* moved horizontally several times during the evolution among Drosophilidae species. Several works provide evidence that *Bari1* HT occurred between *D. melanogaster* and *D. simulans* [53], between *D. melanogaster* and *D. yakuba* [54], and between *D. melanogaster* and *D. sechellia* [52]. Moreover, an additional study in 23 sequenced species of Drosophila showed that horizontal transfer involving *Bari1* elements also occurred between *D. biarmipes* and *D bipectinata* [30]. *Bari1* elements in the two species are nearly identical in sequence, despite that the host species divergence dates back to 27 million years ago [55].

## 4. The Missing Jump to the Next Level

### 4.1. Why We Cannot Use Bari Transposons as Tools for Chromosomal Integration

An initial effort to develop a new chromosomal integration tool based on the *Bari1* transposon was made in Drosophila [56]. A binary transposition system was constructed along the same lines of the *P-element* system: a helper plasmid (the transposase source) and a donor plasmid (the transposon source marked with a white reporter gene), were injected into genotypically suitable fly embryos with the goal of genetically transforming the recipient strain. After the initial excitement due to the identification of few transposition events (i.e., red-eyed individuals), their molecular characterization turned out to be puzzling and frustrating, since the observed phenotype was due to transposition of the *NOF-FB* transposon rather than the transposition of *Bari1* from the donor plasmid. To make a long story short, the expression of the *Bari1* transposase from the helper plasmid has possibly deregulated the *NOF-FB* transposon [56], whose transposition is not currently known and may depend on the activity of unrelated active transposases. Taken together, these fortuitous observations remind us that unpredictable and complex interactions occur when we attempt to manipulate the genome. Following a stress condition, the genome reacts, and the fastest response is often given by the de-repression of transposable elements in several species [57,58,59,60]. We are currently investigating the possible cross-interaction between the overexpression of the *Bari1* transposase and the transposition of NOF-FB in vivo.

Similar attempts to observe the transposition of *Bari* elements through a transposition assay were made using cultured cells as experimental systems. Setting up a binary system, in which the donor transposon was marked with a reporter cassette and the transposase was expressed by a helper plasmid, did not result in a significant integration over the background in Drosophila and human cultured cells [42,61]. Finally, even correcting the diverging aminoacidic residues in critical transposase subdomains, with the guide of a multiple transposase alignment containing other active *Tc1*-like transposons, does not significantly improve the transposition efficiency of *Bari1* in heterologous transposition assays (RMM unpublished observations).

### 4.2. Active or Non-Active? That Is the Question

The reiterated and unsuccessful attempts to develop an efficient transposition system from *Bari* elements led to question whether *Bari* transposons are transposition-competent. Excluding the *Bari2* sub-family, which is entirely composed of non-autonomous elements [39], members of the *Bari1* and *Bari3* sub-families have what it takes to be regarded as functional.

Standing to the general architecture of the *IS630/Tc1/mariner* transposase, the fundamental domains of this enzyme are the DNA binding domain, the GRPR-like domain (which mediates protein–protein interactions), the nuclear localization signal, the homeo-like domain, and the catalytic domain [25]. All these domains can be predicted in the transposase of both *Bari1* and *Bari3*. Furthermore, the functionality of the DNA binding domain has been tested in vitro for both transposons [42,61]. In addition, there is indirect evidence of the transposition ability of *Bari* transposons coming from population genetics studies.

A series of population genetics analyses strongly suggest that *Bari1* is an active transposon in natural populations of *D. melanogaster*. Early studies were performed in 46 populations of *D. melanogaster*, which suggested both *Bari1* inter- and intra-stock polymorphisms [33]. Junakovic and collaborators performed additional studies on a Charolles laboratory population using Southern blot hybridization of single-fly genomic digestions. A strong difference in the insertion pattern can be highlighted in unstable (Charolles) versus stable laboratory strains, suggesting that host factors control the transposition frequency [62] and the insertion preference [63] of *Bari1*. The strongest evidence that *Bari1* is a functional transposon comes from the observation of an excision event in a population established from field-collected flies. The excision event, involving one of the two adaptive *Bari1* insertions in the genome of *D. melanogaster*, was characterized at the molecular level, demonstrating that it was due to genuine transposition (i.e., presence of the transposition footprint) [64].

In conclusion, combining the indirect genetic evidence of the mobility of *Bari1* transposon with the outcome of the experimental transposition assays, we argue that *Bari1* could be tightly regulated, or alternatively we speculate that *Bari1* could be subjected to some unknown types of activation to become transposition-competent. Further and extensive in vitro-directed molecular evolution studies would clarify whether hyperactive variants can be obtained to develop *Bari*-based transposition systems.

## 5. What Do We Know about the Regulation *Bari* Transposons?

The transposition activity of TEs is tightly regulated at various levels. The copy number per haploid genome is a critical factor for the resulting fitness of the whole organism since an excessive TE load can be deleterious for various reasons, ranging from the gene inactivation to the disturbance of the physiological expression networks [13].

Standing to the current knowledge on *Tc1/mariner* elements, two possible types of regulation can be predicted. One is the transposon self-regulation while the other one is the epigenetic regulation.

One mode of self-regulation is exerted through the dissemination throughout the genome of inactive TE copies that still retain the transposase binding ability. Defective copies can act as buffer to titrate endogenous levels of the transposase [40]. Since defective copies of *Bari* elements are abundant, it is conceivable to hypothesize this mode of autoregulation.

There is some experimental evidence in favor of the self-regulation of *Bari* elements through post-transcriptional processing of the transposase mRNA. Few reported data come from overexpression of the transposase in S2R^+^ cultured cells and in *D. melanogaster* embryos [42,61] (Figure 2A). Under these experimental conditions, *Bari1* and *Bari3* transcripts undergo alternative splicing that is potentially translated into a truncated transposase protein. It can be predicted that the transcriptional de-repression of *Bari* elements can inhibit the transposition through the expression of a dominant-negative transposase form. The presence of alternatively spliced transcripts of *Bari1* has also been reported in a *D. melanogaster* HSP83 mutant that deregulates many transposons, including *Bari1* [65,66]. Possibly, the construction of synthetic *Bari1* and *Bari3* transposase genes in which all the conventional, unconventional and cryptic splicing sites have been eliminated, could hopefully enhance the transposition efficiency of both systems.

### Bari Transposons Regulation Relies on the piRNA Pathways

The second mode of regulation of TEs is at the chromatin level. The transcriptional control of TEs is intimately connected to chromatin control at the TE insertion site, and this is possible through the recruitment of specific chromatin remodeling complexes that either open or close the chromatin, thus influencing the transcription.

Genomic structural and transcriptional changes are at the basis of stress responses. In this view, TEs are an amazing source of variability in the short temporal timeframe that allows organisms to promptly react to virtually any kind of stress [67].

Piwi-interacting RNAs (piRNAs) are the most abundant non-coding RNAs in the germline, aiming at the genome safeguard against TE movement [68]. Their action is mainly exerted through the transcriptional silencing. piRNAs usually originate from the so-called piRNA clusters, genomic loci riddled of TE relics that form long non-coding RNAs that are further processed into piRNAs that selectively degrade TE transcripts [69]. Subsequently, a heterochromatic repressive state can be induced at the TE insertion site, reinforcing the transposition control [70,71,72].

In several published reports, piRNA sequences matching *Bari1* and *Bari2* are described [73,74], confirming that *Bari* transposons are among the plethora of Drosophila TEs that are regulated by the piRNA pathway in the germline (Figure 2B). In addition, impairment of the piRNA pathway leads to the transcriptional activation of *Bari1*, such as in the HSP83 mutant [65], suggesting that transcriptional activation occurs upon piRNA depletion.

Despite the lack of experimental evidence, both *Bari* clusters of *D. melanogaster* might support the transcriptional repression of the *Bari* elements. The clusters might indeed act as piRNA clusters, through the expression of small RNA molecules (i.e., piRNA and siRNA) that in turn regulate the transposition frequency.

Several databases offer the opportunity to search in silico annotated Drosophila piRNAs, using sequence similarity criteria. In the piRBase database [75], more than 5800 piRNAs can be retrieved using *Bari1* as a query. Roughly 3800 of them recognize the *Bari1* transposase gene. Notably, a small proportion of piRNAs matching the sequence across the monomer-to-monomer junctions—typical of the *Bari1* heterochromatic clusters—can also be found (Table 1 and Figure 2B). Why a piRNA should be directed against a piece of untranscribed DNA is, however, unclear. Are they byproducts of the piRNA loci transcript maturation or could they have some regulatory or structural role? Time (and further investigations) will tell.

However, direct genetics evidence is currently lacking in support of the piRNA cluster role of the *Bari1* arrays. The currently available chromosomal heterochromatic deletion that removes the *Bari1* cluster in the h39 region also deletes the adjacent *Responder* locus ([32] and references therein; [76]), while deletion only involving one of the main satellites would be more useful. In addition, the lack of precise mapping of the small X-linked cluster currently makes it impossible to perform detailed genetic studies in a *Bari*-cluster null genetic background. Hopefully, such kind of chromosomal aberrations will be soon available due to the application of the most modern genome editing techniques.

## 6. Do Bari Transposons Have a Role in The Genome?

### 6.1. Contribution in Creating Somatic Mosaicism and Adaptive Insertions

It has been reported that TEs are unleashed in some circumstances, to create somatic variability that could have a physiological relevance in certain tissue types [84]. The somatic instability of several TEs has been described in two recent papers. The first paper described the genetic mosaicism of the neurons in the mushroom bodies [85], although this phenomenon has been resized after further studies [86,87]. The second study revealed the somatic transposition in the intestinal stem cells [88,89]. In both cases, *Bari1* was found among the mobilized TEs responsible for the somatic mosaicism in the two tissue types.

The early distribution studies [33] suggested a patchy distribution of *Bari1* euchromatic insertions among 46 different populations of *D. melanogaster* collected worldwide. By contrast, at least two insertions were invariably detected in all the populations analyzed. These insertions occurred at the 55F and 91F bands of the polytene chromosomes. After the completion of the Drosophila genome-sequencing project [90], it appeared clear that both insertions were intimately connected to host genes. Indeed, the insertion in the 55F region falls near the Juvenile hormone epoxy hydrolase (*Jheh)* gene cluster (*Bari-Jheh* insertion), while the 91F insertions are associated to the *cyp12a4* gene (*Bari1-Cyp12a4* insertion). Further studies in which ChIP-seq data were integrated with gene expression analyses and phenotypic assays demonstrated that the *Bari-Jheh* insertion introduced extra antioxidant response elements upstream of *Jheh1* and *Jheh2* genes [91]. Furthermore, *Bari-Jheh* is differentially associated to H3K27me3 in stress vs. non-stress oxidative conditions, suggesting the addition of histone marks to the intergenic region between *Jheh2* and *Jheh3* genes, and its association with histone marks enrichment in the promoter of *Jheh1* gene [92].

The *Bari1-Cyp12a4* insertion overlaps with the 3′ end of the *Cyp12a4* gene and it contributes to the stability of the mature transcript. It has been demonstrated that *Bari1-Cyp12a4* supplies the polyadenylation sequence to the *Cyp12a4* transcript [64].

In addition, the *Bari1-Cyp12a4* showed an enrichment of H3K27me3 under oxidative stress conditions [92], suggesting an active role of the insertion in altering the epigenetic context in response to stress.

### 6.2. Possible Role in Chromatin Assembly

TEs can also act as potent epigenetic modifiers that could change both gene expression and chromatin structure [93] with important evolutionary implications [94]. This is possible thanks to the ability to recruit chromatin proteins [95].

Large-scale mapping of in vivo binding sites of chromatin proteins using tethered dam methyltransferase [96,97] showed that the clustered *Bari1* elements in the h39 region of the 2nd chromosome are targets of HP1, a chromatin protein mainly involved in heterochromatin formation [98]. It has been reported that *Bari1* also contains Polycomb responsive elements that could collaborate in recruiting additional heterochromatin components [91]. This could be of particular relevance when clustered copies of *Bari1* are exploited in the genome. It is therefore possible that both *Bari1* clusters could have acquired a structural role in the heterochromatin of *D. melanogaster*, such as to ensure proper chromosome conformation and stability over the cell cycle.

## 7. The Blurry Promoter: Who Wants to Live Forever?

**Figure 3:**
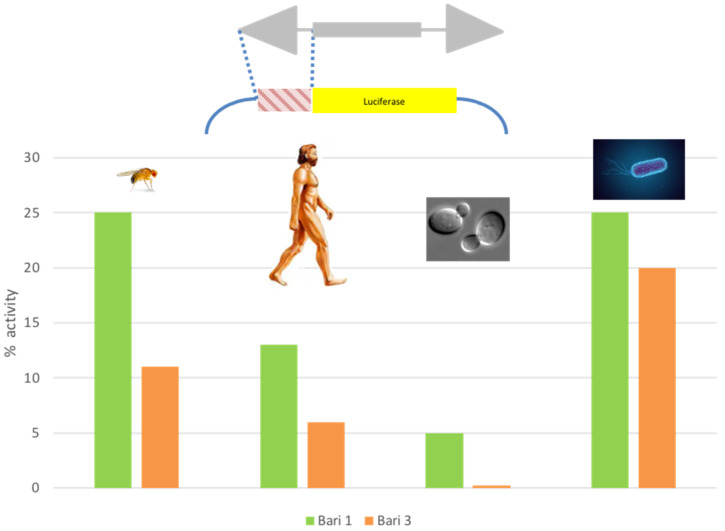
Activity of the native promoter of *Bari1* and *Bari3* in heterologous cellular systems. The transposon fragment tested in promoter-luciferase assays is depicted in the upper part of the figure. The % activity compared to strong species-specific constitutive promoters (SV40, copia, URA3, and CAT for human, fly, yeast, and bacteria cells, respectively), arbitrarily assumed as 100%, is reported on Y axis of the chart.

Along with the characterization of the *Bari1* transposition system, the strength of the *Bari1* promoter was tested. Luciferase promoter assays [99] conducted in fly cell cultures (S2R^+^) revealed that *Bari1* has a weak promoter if compared to the strong constitutive *copia* promoter [100]. Weak promoters are typical of the *Tc1*/*mariner* transposons, which suffer from overproduction inhibition and therefore tend to limit the amount of transposase [101]. Unexpectedly, the *Bari1* promoter drives the reporter transcription also in human, bacteria, and yeast cells [100]. This is quite counterintuitive, since evolutionarily distant genomes (e.g., animal and bacteria) have profoundly different modes of transcription and promoter organization [102]. Surprisingly, the promoter of *Bari3* also showed a similar promiscuous activity (Figure 3). Due to this feature, the promoters were named “blurry”, to highlight that they do not “sharply” activate the transcription in a single or few closely related species. These findings led to the hypothesis that the members of the *Bari* family can take advantage of this feature to engage successful HTT events. The ability of TEs promoter to sustain the transcription of associated genes upon HTT is recognized as one of the bottlenecks that restricts the success of HTT [103]. The presence of blurry promoters was also assessed in other *Tc1*- and *mariner*-like TEs. This observation is in agreement with the known predisposition of *Tc1*/*mariner* elements to be horizontally transferred [50,104]. It also suggests a common origin of this promoter type, and a possible common survival strategy for the elements belonging to the *Tc1/mariner* superfamily [105]. The discovery of the blurry promoters in the *Bari* family has also opened the possibility to implement them, together with many other TE-derived transcriptional control sequences, into expression modules in order to expand the repertoire of the existing expression vectors with a considerable improvement of their performances [4].

## 8. Concluding Remarks

### What We Have Learned and What We Still Have to Learn from Bari Transposons

In this review, we summarized the current scientific literature concerning the transposable elements belonging to the *Bari* family. Although the scientific interest around this transposon was mainly focused on its evolutionary history, several important experimental outcomes are worth noting, which have been (and hopefully will be in the future) important to deepen our understanding of TE biology.

Many aspects of the *Bari* transposons’ biology are still not completely known and should be clarified, such as the low transposition rate in vitro. Studies on wild-collected fly populations will likely be the key to obtaining insights into the regulation of the *Bari* elements, as suggested by studies of Junakovic et al. [62].

It is possible that, as suggested by different studies, the *Bari* transposase could be intrinsically error-prone, and only occasionally it gives rise to productive transposition events. Fixing the transposase could possibly allow to obtain a robust transposition system. We still have to understand how the blurry promoter has evolved, and how it can be used to develop new tools for the genetic manipulation.

Finally, the determination of the structural and functional role of the heterochromatic clusters is a task that deserves further investigations, and will be accomplished with the aid of the genome editing technologies.

We think that more secrets associated to TEs remain to be discovered, and the continuous effort in dissecting the most diverse TEs in nature will contribute to clarifying the role of these genetic elements in evolution, hopefully attracting more interest in the field of TE biology, especially from emerging scientists.

## Figures and Tables

**Table 1:** piRNAs targeting the Bari1 inter-monomer junction. Data extracted from the piRBase database [75] (last accessed March 2021). The sequence used to query the database encompasses an inter-monomer junction of the heterochromatic *Bari1* cluster (TTTGACCACCTCTGGTCATGGTCAAAATTAT). Sequence matching either the left or the right monomers are marked with different colors (blue and red, respectively). O = ovaries; F = follicle cells; E = eggs; W = wild type; T = transgenic; M = mutant.

Name	Tissue	Genetic Background	Methods	Reads	Sequence	Length	Reference
piR-dme-2858217	O; E	W; T; M	oxidized small RNA small RNA	1–3	TTTGACCACCTCTGGTCATGGTCAAAA	27	[77,78,79]
piR-dme-3826713	O	W; T; M	small RNA oxidized small RNA	1–6	TCTGGTCATGGTCAAAATTATTTT	24	[77,79,80,81]
piR-dme-8496440	O	T	small RNA	1–3	TTTGACCACCTCTGGTCATGGTCAA	25	[80]
piR-dme-13381112	O	T	small RNA	1	CCACCTCTGGTCATGGTCAAAATTAT	26	[80]
piR-dme-21388569	F; O	W; T	small RNA	1–9	TTTGACCACCTCTGGTCATGGTCAAAAT	28	[79,81,82]
piR-dme-21631816	O	W	small RNA	1	CACCTCTGGTCATGGTCAAAATTAT	25	[83]
piR-dme-26496558	O	T	small RNA	1	ACCACCTCTGGTCATGGTCAAAATTA	26	[79]
piR-dme-26779428	O	T	small RNA	1	CCACCTCTGGTCATGGTCAAAAT	23	[79]
piR-dme-27814712	O	T	small RNA	1	TCTGGTCATGGTCAAAATTATTT	23	[79]
piR-dme-29438648	O	T	small RNA	1	TGACCACCTCTGGTCATGGTCAAA	24	[79]
piR-dme-29670403	O	T	small RNA	1	TTGACCACCTCTGGTCATGGTCAAAA	26	[79]
piR-dme-30537191	O	T	small RNA	2	TTTGACCACCTCTGGTCATGGTCAAA	26	[79]
piR-dme-31705044	O	T	small RNA	1	CTCTGGTCATGGTCAAAATTATTT	24	[79]
piR-dme-32470189	O	W	small RNA	1	TTTGACCACCTCTGGTCATGGTCA	24	[81]
piR-dme-33774286	O	W	small RNA	1	TGACCACCTCTGGTCATGGTCAAAAT	26	[81]
piR-dme-38817646	O	T	small RNA	1	CTCTGGTCATGGTCAAAATTATTTT	25	[81]

## Data Availability

Not applicable.
